# Ubiquitination Regulators Discovered by Virtual Screening for the Treatment of Cancer

**DOI:** 10.3389/fcell.2021.665646

**Published:** 2021-05-12

**Authors:** Ying-Qi Song, Chun Wu, Ke-Jia Wu, Quan-Bin Han, Xiang-Min Miao, Dik-Lung Ma, Chung-Hang Leung

**Affiliations:** ^1^State Key Laboratory of Quality Research in Chinese Medicine, Institute of Chinese Medical Sciences, University of Macau, Taipa, Macau; ^2^Department of Chemistry, Hong Kong Baptist University, Kowloon, Hong Kong; ^3^School of Chinese Medicine, Hong Kong Baptist University, Kowloon, Hong Kong

**Keywords:** ubiquitination regulator, virtual screening, drug discovery, cancer, treatment

## Abstract

The ubiquitin-proteasome system oversees cellular protein degradation in order to regulate various critical processes, such as cell cycle control and DNA repair. Ubiquitination can serve as a marker for mutation, chemical damage, transcriptional or translational errors, and heat-induced denaturation. However, aberrant ubiquitination and degradation of tumor suppressor proteins may result in the growth and metastasis of cancer. Hence, targeting the ubiquitination cascade reaction has become a potential strategy for treating malignant diseases. Meanwhile, computer-aided methods have become widely accepted as fast and efficient techniques for early stage drug discovery. This review summarizes ubiquitination regulators that have been discovered via virtual screening and their applications for cancer treatment.

## Introduction

Ubiquitin is a 76-residue protein that is highly conserved in most eukaryotes (Finley and Chau, 1991; Ben-Neriah, 2002). The main function of ubiquitin is to mark proteins that need to be broken down and hydrolyze them using the ubiquitin-proteasome system (UPS) (also known as the ubiquitin-proteasome pathway, UPP) (Weissman, 2001). The C-terminus of ubiquitin contains the functional for ligation to acceptor proteins (Rotin et al., 2000; Peng et al., 2003). The ubiquitin-attached proteins are directed to the proteasome for breakdown (Voges et al., 1999). Ubiquitin can also label transmembrane proteins, such as receptors, to remove them from the cell membrane (Foot et al., 2017). The process by which target proteins are labeled with ubiquitin is called ubiquitination, and it is one of the most common forms of post-translational protein modifications (Popovic et al., 2014). Besides ubiquitin itself, ubiquitin-like molecules such as SUMO, NEDD8, or ISG also serve to regular protein homeostasis in cells (Ritchie and Zhang, 2004; Zhong et al., 2012b; Liu and Nussinov, 2013; Gâtel et al., 2020).

**FIGURE 1:**
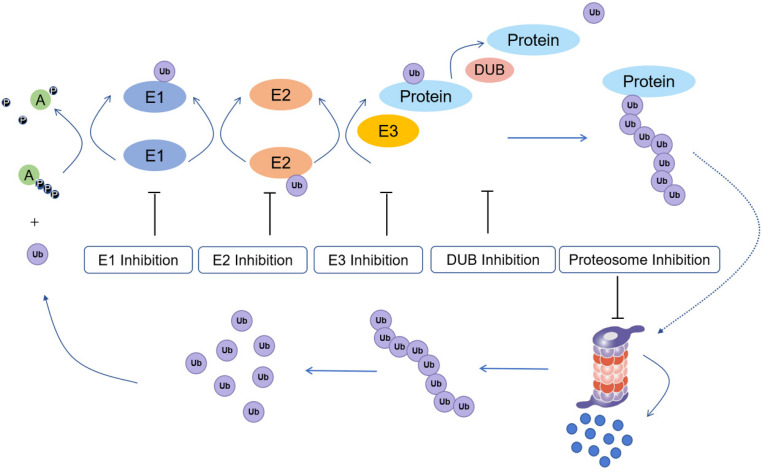
Ubiquitination process and potential drug inhibition targets (E1: ubiquitin-activating enzyme; E2: ubiquitin conjugating enzyme; E3: ubiquitin protein ligase; Protein: substrate/target protein; DUB: deubiquitinating enzyme).

The process of ubiquitination includes the sequential actions of three main enzymes (Figure 1): ubiquitin-activating enzymes (E1s), ubiquitin-conjugating enzymes (E2s), and ubiquitin protein ligases (E3s) (Fajerman et al., 2004). E3 enzymes can be further subdivided into RING (really interesting new gene) family and HECT (homologous to E6-APC terminus) family proteins (Uchida and Kitagawa, 2016). In the mechanism of ubiquitination, the C-terminus of ubiquitin is first activated for nucleophilic attack activated by E1s. Then, the activated ubiquitin is transferred onto E2s. Finally, E3s transfer the activated ubiquitin from E2s to the lysine residue of a substrate protein (Rape, 2018). Monoubiquitination is mainly involved in the regulation of endocytosis, meiosis, chromatin remodeling and lysosomal targeting, while polyubiquitination is involved in targeted modification of proteins to achieve proteasome degradation, DNA repair and immune signaling (Sun and Chen, 2004).

Ubiquitination has a central function in protein regulation and mediates various cellular processes, such as DNA repair, cell cycle control, apoptosis, the inflammatory response, and antigen presentation (Pickart, 2001; Zhuo et al., 2015). However, dysregulated ubiquitin-dependent proteolysis can result in pathological events, including cancer (Baarends et al., 1999; Saffari-Chaleshtori et al., 2020). Hence, targeting ubiquitination regulators is an emerging approach for the treatment of cancer. E1s regulate all downstream ubiquitination reactions, thus targeting E1s could potentially affected the ubiquitination status of downstream tumor-related proteins. E2 inhibitors offer higher potential selectivity for anticancer therapy. E3s specifically recognize protein substrates, thus making it a target for a variety of cancer therapies. Besides E1, E2, and E3 inhibitors, proteasome inhibitors have also been developed as anticancer agents. Finally, deubiquitinating enzymes (DUBs) have also been studied as anticancer targets due to their role in mediating the stability of proteins.

Fluorescence resonance energy transfer (FRET), homogeneous time-resolved fluorescence (HTRF), dissociation-enhanced lanthanide fluoroimmunoassay (DELFIA), electrochemiluminescence (ECL)-based assay, scintillation proximity assay (SPA), and laboratory-based *in vitro* and *in vivo* ubiquitination assays are widely used assays for screening ubiquitination inhibitors (Sun, 2005). However, each of these methods may have their own distinct disadvantages, such as low-throughput, high-cost, or susceptibility to background interference. Meanwhile, virtual screening has recently emerged as an alternative approach to identify ubiquitination regulators. Virtual screening is usually described as a computational algorithm using cascading sequential filters that can narrow the set of potentially biologically active lead-like hits against predetermined drug targets. Since “testing” is performed *in silico*, virtual screening does not consume valuable materials (Lavecchia and Di Giovanni, 2013). Subsequently, those companies identified as potential inhibitors by virtual screening can be verified using *in vitro* experiments (Walters et al., 1998; Shoichet, 2004). With continual advances in computing power, virtual screening is increasingly utilized as a supplement to high-throughput screening to enhance the rapidity and effectiveness of the drug discovery and development program (Good et al., 2000).

Recently, virtual screening has been employed for identifying inhibitors of cancer targets (Zhong et al., 2016; Li et al., 2020). For example, Russo Spena et al. (2019) identified VS10 as a selective PIN1 inhibitor with anti-ovarian cancer activity through virtual screening. Utomo et al. (2012) used virtual screening to identify drug-like compounds that can interrupt the stability of the p53-mortalin complex interaction, an anticancer target. Yousuf et al. (2017) discovered five anti-breast cancer multi-target inhibitors via structure-based virtual screening and molecular docking methods. Pan et al. (2013) based on a combination of ligand-based virtual screening and experimental testing, identified 19 drugs that have significant inhibitory effects on breast cancer resistance protein transport. Virtual screening has also been applied to the study of ubiquitination regulators. Hirayama et al. (2007) identified three compounds with similar basic dihydropyrrole skeletons as DUB (UCH-L3) inhibitors through virtual screening. Pirolli et al. (2019) identified E3 ubiquitin protein ligase NEDD4-1 inhibitors based on a virtual screening method to restore the level of Spry2 in cancer cells.

Compared to the application of virtual screening technologies against other anticancer targets, their use for identifying ubiquitination regulators remains underreported. In this review, we outline different virtual screening methods and highlight the latest developments in the use of virtual screening techniques for discovering ubiquitination regulators (Table 1). The important role of virtual screening technology for the discovery of ubiquitination regulators in the context of anticancer treatments is also described.

**TABLE 1:** Ubiquitination regulators identified by virtual screening.

	Compound	Structure	Target	Residues involved in interaction	Method	Verified activity	References
**E1**	**1** (LZ3)	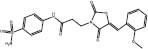	NAE	Ile148/Asp100/Gly79	SBVS	*In cellulo*	Zhang et al., 2014

	**2**	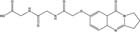	NAE	Asp100/Lys124/Asp167/Gln149	SBVS	*In cellulo*	Zhong et al., 2012a

	**3**	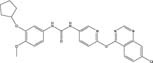	SUMO E1	Arg21/Asn56/Arg59/Gln60/Lys72	SBVS	*In vitro*	Kumar et al., 2013

**E3**	**4** (S01)	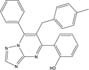	MDM2-p53	Leu54/Leu57/Gly58/Ile61/Met62/Val193/His96/Ile99/Tyr100	SBVS	*In cellulo*	Atatreh et al., 2018

	**5** (S02)	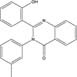	MDM2-p53	Leu54/His96/Val193/Ile99	SBVS	*In cellulo*	Atatreh et al., 2018

	**6**	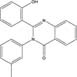	Skp2	Trp97/Asp98	SBVS	*In cellulo*	Chan et al., 2013

	**7**	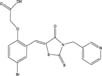	Skp2	Q52/R44/R344	SBVS&LBVS	*In cellulo*	Wu et al., 2012

	**8**	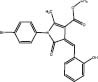	Skp2	Q52/R44/R344	SBVS&LBVS	*In cellulo*	Wu et al., 2012

	**9**	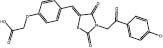	Skp2	R294/R44	SBVS&LBVS	*In cellulo*	Wu et al., 2012

	**10**	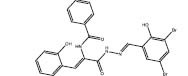	Skp2	R294/R44	SBVS&LBVS	*In cellulo*	Wu et al., 2012

**Proteasome**	**11**	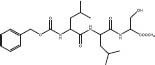	proteasome	Thr1/Arg19	SBVS	*In cellulo*	Di Giovanni et al., 2016

	**12** (G4-1)	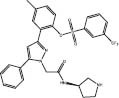	proteasome	Asp114/Ala20/Val31/Ala49/Lys33/Gly47/Thr1	SBVS	*In cellulo*	Miller et al., 2015

**DUB**	**13**	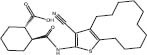	USP7	Val296/Arg408/Phe409	SBVS	*In cellulo*	Liu S. et al., 2020

	**14**	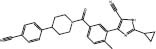	UCHL-3	Leu168/Leu55/Ala11/Pro8/Val166/Asn12	SBVS	*In vitro*	Alakhdar et al., 2020

	**15**	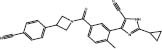	UCHL-3	Leu168/Arg221/Leu55/Thr157/Pro8/Glu10/Val166	SBVS	*In vitro*	Alakhdar et al., 2020

	**16**	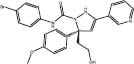	UCHL-3	Leu55/Pro160/Leu168/Val166/Ala11/Thr157/Pro8	SBVS	*In vitro*	Alakhdar et al., 2020

## Ubiquitination Process and Function

### Ubiquitination and the Enzymes Involved

The UPS regulates many eukaryotic signaling pathways through controlling the quick and timely degradation of transcription regulators, enzymes and other proteins in the cell (Nandi et al., 2006). The UPS also has an important role in preventing the accumulation of misfolded or harmful proteins (Ciechanover, 1998). Ubiquitin attachment involves the formation of an isopeptide bond between the glycine C-terminus of ubiquitin and the lysine NH_2_ group of the substrates (Komander and Rape, 2012; Rieser et al., 2013). E1s, E2s, and E3s participate in a series of biochemical reactions in order to covalently bind ubiquitin to the substrate. Protein ubiquitination is also a highly reversible process. In some cases, DUBs can remove ubiquitin from protein substrates, thereby protecting the protein from degradation and releasing free ubiquitin for recycling (Kim et al., 2003). However, in other cases, DUBs also enhance substrate degradation.

Ubiquitination substrates can be monoubiquitinated or polyubiquitinated at one or multiple lysines (Sadowski et al., 2012). The manner of modification can depend on the position of the lysine (K6, K11, K27, K29, K33, K48, K63) or *N*-terminus methionine of ubiquitin is connected to the end of ubiquitin, resulting in different chain types (Zhou et al., 2014). Critically, the kind of ubiquitin signal governs the physiological consequences of these changes. For instance, K48 and heterotypic K11/K48 chains usually target the 26S proteasome to degrade substrates (Grice et al., 2015; Zheng and Shabek, 2017), whereas chains connected through K6, K27, K33, K63 often have non-proteolytic purposes (Grice and Nathan, 2016). These modifications are read by proteins with ubiquitin-binding domains, which identify chain-specific residues at the ubiquitin ends and the junction region that connects the two ubiquitin molecules. Many proteins dynamically bind to single or multiple ubiquitin molecules or chains, usually determining the half-life, location, or function of the protein (Tenno et al., 2004).

Besides its proteolytic effects, ubiquitination also controls a wide range of non-proteolytic functions, including regulation of DNA repair, enzyme activity, inflammatory signals, autophagy, receptor internalization, protein complex assembly, and intracellular trafficking (Welchman et al., 2005). For example, the control of membrane protein types and abundance is usually regulated by ubiquitination (Hicke and Dunn, 2003). In this process, ubiquitination is a signal to classify, transport, and remove membrane proteins (such as ion channels, transporters, and signal receptors) through endocytosis (MacGurn et al., 2012). Ubiquitination also participates in the shedding of membrane-associated proteins, thus also controlling their potential transport to adjacent cells.

Aberrant ubiquitin signaling is considered to be the molecular cause of certain cancers, neurodegeneration, cardiovascular or immune diseases. Dysregulated ubiquitination may cause abnormal activation or inactivation of pathways resulting in oncogenesis or defects in cell metabolism pathways. Improper or insufficient assembly of protein complexes are associated with inflammation or aberrant DNA repair activity, while the buildup of misfolded proteins within the endoplasmic reticulum or cytoplasm is a hallmark of neurodegenerative diseases. Any of these changes will cause damage to cell function.

### Ubiquitin-Like Modifiers

Recently, it has been discovered that ubiquitin can also be phosphorylated, acetylated and modified by binding to ubiquitin-like proteins, which indicates that the process and its regulation are much more complicated than originally thought (Swatek and Komander, 2016). Similar to the process of ubiquitination, small ubiquitin-related modifier (SUMO) be attached to the lysine moieties of proteins, thus regulating their localization, stability, and interactions (Johnson, 2004). At present, the most studied ubiquitin modification system that can compete with ubiquitination for the modification of Lys residues is SUMO. The SUMO system is simpler than the ubiquitin system. It only has a single E1 enzyme, a single E2 enzyme and a few E3 enzymes.

The other two common ubiquitin modifiers are NEDD8 (neural precursor cell-expressed developmentally downregulated 8) and ISG15 (interferon-stimulated gene 15) (Xirodimas et al., 2004). NEDD8, a 81-amino acid protein with 60% identity and 80% similarity to ubiquitin, is highly conserved in eukaryotes (Kamitani et al., 1997; Zhong et al., 2015). Neddylation can extensively regulate biological events, such as cell cycle, signal transduction and immune recognition. Enzymes that regulate neddylation include NEDD8-activating enzyme, NEDD8-binding enzyme and NEDD8 ligase. Similar to ubiquitination, neddylation is also a dynamic modification process that a maintains balance between neddylation and deneddylation.

ISG15 can also covalently modify proteins, but does not mediate protein degradation (Morales and Lenschow, 2013). It is currently believed that ISG15 modification is mainly involved in the regulation of the innate immune function through leukocyte chemotaxis and the process of interferon action. ISG15 may also play an important role in stimulating cell proliferation and enhancing cytotoxicity.

## Ubiquitination Regulator Discovery by Virtual Screening

Virtual screening is typically performed in hierarchical workflow with sequential steps (each with their own advantages and limitations) that filter and remove unwanted molecules. Molecules that pass all stages of the virtual screening are called the “hit compounds.” One method of sorting the hit compounds is clustering, which is an unsupervised learning technique were input data is fed into the algorithm in order to identify patterns and classify the data into several categories. By using clustering to group hits based on their structure, the biased choice of molecules can be circumvented and representative samples of compounds can be obtained. Hierarchical clustering, HDBSCAN and k-means clustering are different clustering methods for hit selection (Gimeno et al., 2019). Finally, hit compounds must also be verified through experiments to validate their bioactivity.

Recently, virtual screening has become a powerful method to supplement the existing array of high-throughput screening platforms. Using computer-assisted virtual screening, potential hits can be quickly identified *in silico* to decrease the number of molecules needed to be tested *in vitro* and *in vivo* (Ma et al., 2013). Integrating virtual methods into the development of pharmaceutical leads can significantly decrease the economic cost of synthesis or biological experiments (Yang et al., 2016).

**FIGURE 2:**
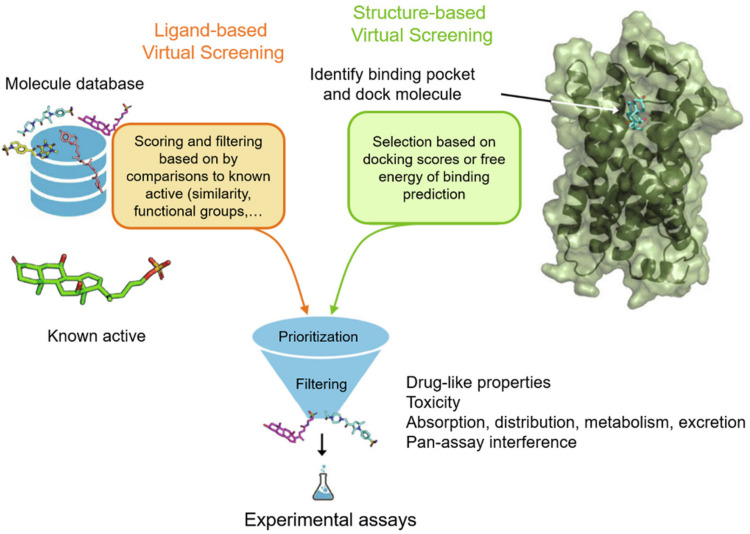
Workflow for ubiquitination regulator discovery by ligand-based virtual screening (LBVS) and structure-based virtual screening (SBVS). Reproduced, with permission, from Reference Raschka and Kaufman (2020).

Virtual screening protocols can be broadly classed into two categories: structure-based virtual screening (SBVS) and ligand-based virtual screening (LBVS) (Figure 2; Muegge and Oloff, 2006). SBVS, which includes docking, needs a 3D structure of the protein target (Stahura and Bajorath, 2005). In docking, candidate ligands are docked into the target protein, and a scoring function is applied to evaluate the possibility of high-affinity binding between the ligands and proteins. On the other hand, LBVS techniques generally use a library of ligands with known activities (Ripphausen et al., 2011). LBVS uses active compounds as templates, then searches chemical molecular structures that matches the shape or pharmacophore model of the compound. SBVS and LBVS strategies will be discussed individually in more detail in the sections below.

### Structure-Based Virtual Screening (SBVS)

In SBVS, the three-dimensional structure of the protein is employed. SBVS aims to identify compounds with unknown affinity to the target from their three-dimensional structure (Figure 2; Lionta et al., 2014). The process of SBVS usually starts with the information of the three-dimensional target structure of interest (Kroemer, 2007). This structure can be obtained from experimental data such as X-ray crystallography, nuclear magnetic resonance, or neutron scattering experiments, or can be constructed from homology modeling or molecular dynamics simulations. When performing SBVS, the druggability of the receptor, the selection of the most relevant protein conformation, and the flexibility of the receptor, should also be considered. After the target model is prepared, the compound library also has to be preprocessed in order to assign the proper stereochemistry, tautomeric, and protonation states.

### Ligand-Based Virtual Screening (LBVS)

Unlike structure-based methods, LBVS starts with known active ligands as templates (Figure 2). Generally speaking, the LBVS method relies on the use of structural or pharmacophoric descriptors, and analyzes the relationship between active compounds and databases or test compounds (Chen et al., 2007). Many different types of molecular descriptors have been developed, and they have been extensively reviewed elsewhere (Shahlaei, 2013). However, one limitation of ligand-based methods is that they limit the diversity of hits, because they are mainly biased toward the properties of known ligands (Baber et al., 2006). Hence, newer LBVS methods that identify remote similarity connections, that is, compounds that are structurally different from templates but possess similar activities, are being developed. The identification of such compounds is often critical to avoid lead optimization bottlenecks.

## Ubiquitination Regulators for Cancer Treatment

There is increasing evidence that the UPS plays a key role in tumorigenesis. Ubiquitin-mediated signals are often altered in cancer cells, including dysregulation of tumor suppressors and oncogenes. For example, the tumor suppressors p53 and p27 can be regulated by ubiquitination (Thibaudeau and Smith, 2019). Hence, the use of proteasome inhibitors could alter balance between pro-apoptotic proteins and anti-apoptotic proteins, and induce cell cycle arrest and apoptosis. Moreover, targeting specific ubiquitin pathway enzymes (E1s, E2s, and E3s) and DUBs could be a method of developing more selective anti-tumor drugs with better toxicity characteristics than proteasome inhibitors such as bortezomib (Mattern et al., 2012).

Meanwhile, virtual screening is an efficient method to screen large libraries of compounds against a pharmacological target *in silico*. In this section of the review, we highlight examples of ubiquitination regulators that have been discussed by virtual screening.

### Identification of E1 Regulators by Virtual Screening

Inhibitors of E1s are a potentially new class of cancer drug (Yang et al., 2007). Two types of ubiquitin-activating E1 enzymes are known: the main is UBA1, while the newly discovered UBA6 currently has unclear functions. However, due to the lack of specificity of E1s, there are few reports on E1 inhibitors discovered by VS.

Like the ubiquitination pathway, NEDD8 requires activation by the E1-like NEDD8-activating enzyme (NAE). NAE specifically regulates the degradation of substrates of the cullin-ring ubiquitin E3 ligases, and is a potential target for anticancer drugs (Zhong et al., 2014; Wu et al., 2018).

Zhang et al. (2014) adopted a combined strategy of VS to discover diverse covalent inhibitors of NAE, involving docking-enabled pharmacophore model (according to the possible active conformation of the selected covalent inhibitors) and a dynamic structure-based pharmacophore (derived from snapshots of molecular dynamics simulations). The ZINC publicly database containing over 22 million compounds was initially screened with the Align to Selected Substructure module in Discovery Studio 2.5. Conformations of the focused library of 27,996 molecules were then generated using the Build 3D Database utility, which were screened using the Search 3D Database (for ligand-based pharmacophore screening) or Screen Library (for structure-based pharmacophore screening) modules to produce 256 highly ranked hits. Next, covalent docking was performed using Gold 5.0, resulting in eight hits, of which three were confirmed to be active after bioassay evaluation. Compound **1** (LZ3) was determined to be the most effective NAE inhibitor, with IC_50_ = 1.06 ± 0.18 μM. Moreover, a cell-based assay validated the proposed covalent model for compound **1**. MTT assay results showed that compound **1** strongly inhibited the proliferation of Caco-2, Bcl-7402 and MCF-7 cancer cells, with IC_50_ values of 12.3 to 29.5 μM.

Zhong et al. (2012a) used SBVS of from the ZINC of natural products database of over 90,000 compounds to discover a dipeptide-conjugated deoxyvasicinone compound (**2**) as an NAE inhibitor. The Internal Coordinate Mechanics was used for docking, where flexible ligands were matched to a grid representation of the protein and scored based on the predicted binding affinity. Nine top-scoring ligands were obtained and tested in a preliminary E1 NAE activity assay, and four of these showed inhibitory effects against Ubc12-NEDD8 conjugation *in vitro* with the most potent of these being compound **2**. Compound **2** was hypothesized to act via reversibly binding to the ATP-binding domain. Compound **2** inhibited NAE activity with micromolar potency in both cell-based and cell-free assays.

SUMO E1 is also a potential therapeutic target for many human diseases including cancer. In order to better identify the SUMO E1 small molecule inhibitors with drug-like properties, Kumar et al. (2013) performed virtual screening against SUMO E1 using the Maybridge small molecule library. Nearly 78,000 molecules in the Maybridge library were docked against SUMO E1 using a two-step docking strategy. First, ligands with non-ideal energetics and geometries were removed using Autodock-Vina. The top-ranked molecules from the initial stage were then re-docked using RosettaLigand, which uses three-dimensional conformations of ligands. Hits were ranked using the RosettaLigand energy function as well as ligand-protein interface scores, followed by prioritization of the highest-scoring docking hits using molecular mechanics. Then, a similarity search against the ZINC database identified a series of new compounds via “scaffold hopping.” Subsequent *in vitro* testing using revealed that compounds containing a quinazolinyloxy biaryl urea were new inhibitors of SUMO E1, with the most potent compound **3** of this class having an IC_50_ of 14.4 ± 1.3 μM against SUMO E1. This compound acts by reducing the SUMO E1-SUMO thioester bond formation. By employing virtual screening in conjunction scaffold hopping, they also found that compounds containing pyrazole and thiazolium urea moieties can function as moderate inhibitors of SUMO E1 (Kumar et al., 2016).

### Identification of E2 Regulators by Virtual Screening

As an intermediate between the E1 and E3 proteins, the E2s play an important function in determining the kind of polyubiquitin chain connected (Rodrigo-Brenni and Morgan, 2007). At present, about 40–50 genes encoding ubiquitin-conjugating enzymes have been found in the human genome, which is much greater than the number of E1 enzymes. After E2s bind to the activated ubiquitin, ubiquitin molecules are delivered to ubiquitin ligase E3 (Gundogdu and Walden, 2019). Because each E2 can only associate and cooperate with a specific set of E3 enzymes, targeting the E2–E3 interaction could be a potentially selective anticancer approach. However, E2 enzyme inhibitors are currently only in the developmental and pre-clinical testing stage as they still lack sufficient specificity for E2s.

Cdc34 is an E2 enzymes directly related to oncogenesis. Arrigoni et al. (2014) utilized virtual screening and docking to screen over 735,000 compounds from the ZINC database. In the first stage, coarse molecular selection was performed by shape complementarity using DOCK Blaster. The top 500 ligands from the initial stage were re-docked against Cdc34 using Autodock version 4.2, followed by a filtering step involving spatial criteria and binding free energy to shortlist 20 hit molecules. Common structural features of these 20 molecules could be employed as pharmacophoric elements for future investigations.

The SUMO E2 conjugating enzyme Ubc9 is highly expressed in various human cancer cell lines, suggesting that Ubc9 may be an attractive drug target (Tomasi et al., 2012). Duan et al. (2009) used SBVS to screen small chemical compounds as potential anticancer drugs by determining the structure and potential target sites of Ubc9. They analyzed the interface regions between Ubc9 and its binding partners, in order to identify possible targeting sites on Ubc9 that can be used for virtual screening and ligand design.

### Identification of E3 Regulators by Virtual Screening

The E3s are responsible for the direct ligation of ubiquitin to the protein, thus conferring substrate specificity and selectivity. Mutations or down-regulation of E3 enzymes can often be detected in different tumors (Shafique et al., 2018). As the substrate recognition component of the UPS pathway, there are about 500–1000 ubiquitin ligases in the human body (Ottis et al., 2017). Hence, targeting specific E3 ligases will only affect a particular subset of ubiquitin substrates, without affecting for the entire ubiquitination pathway. As a result, E3 enzymes have great potential for cancer treatment.

The Von Hippel-Lindau (VHL) E3 ubiquitin ligase controls the ubiquitination and subsequent of its substrate, hypoxia inducible factor 1 (HIF-1). VHL is a potential target for a variety of diseases, including anemia, inflammation, neurodegenerative diseases and cancer. Liu Y. et al. (2020) employed a combined ensemble-based and ligand-based virtual screening strategy to finding potential inhibitors against VHL from the Specs database. The use of ensemble-based virtual screening to distinguish the active molecules from inactive molecules increases precision compared to usual single SBVS methods. Ten representative molecules were obtained from the virtual screening, and the predicted binding modes of the first five molecules were analyzed with comparison to the reference ligand. However, further *in vitro* and *in vivo* experiments are needed to verify the effectiveness of the ten candidates.

MDM2 protein regulates p53 activity by acting as a ubiquitin E3 ligase (Brooks and Gu, 2006). MDM2 can bind to the transcriptional activation domain of p53, inhibit its transcriptional activity, promote its ubiquitination and degradation, and hence block the functions of p53-regulated cell cycle stagnation and apoptosis induction (Aydin et al., 2020). Nutlin 3a was the first small molecule inhibitor targeting MDM2 that acts through binding to p53 in order to block its interaction with MDM2, thereby increasing the level of p53 in cells.

Atatreh et al. (2018) identified compounds **4** (S01) and **5** (S02) as MDM2 inhibitors through pharmacophore and structure-based *in silico* screening. First, they constructed a pharmacophore from the p53-binding pocket of the Mdm2 protein (PDB: 3JZK) using three pharmacophore elements: one hydrophobic/aromatic, one hydrophobic, and one aromatic. This pharmacophore was used to preliminarily screen over 580,000 molecules from the TimTec Compound Library. Compounds that matched the pharmacophore were then docked against the targeted protein using GLIDE. The highest-ranked 500 ligands were manually inspected for their binding pose within the p53 binding site of Mdm2, after which 40 were chosen for *in vitro* Mdm2-p53 inhibition using ELISA. A few hits showed comparable potency to Nutlin-3a at inhibiting Mdm2-p53, including S01 (**4**) and S02 (**5**). Compound **4** binds Gly58 at the active site of MDM2, which is a non-canonical interaction observed by MDM2 co-crystallization inhibitors that blocks the interaction of MDM2-p53. These compounds showed potential anticancer activity against breast cancer cell lines of different subtypes.

Skp2 E3 ligase is overexpressed in a number of cancers, and it has functions in cell metastasis, cycle, metabolism, senescence, and cancer progression (Chan et al., 2010). Chan et al. (2013) performed SBVS on 120,000 commercial compounds using HiPCDock. Hits were chosen based on both calculated binding strength as well as other drug-like characteristics including molecular mass and solubility. The top 25 hits were then tested using an *in vitro* pull-down assay to Skp2 and Skp1 inhibition. From the screening campaign, compound **6** emerged as a potent and specific Skp2 inhibitor. Compound **6** displayed potent antiproliferative activity against prostate cancer cell lines, including PC-3 (IC_50_ = 5.61 μM) and LNCaP (IC_50_ = 1.22 μM), but only had slight effects on normal prostate epithelial (PNT1A) cells. Finally, this compound showed powerful *in vivo* antitumor activity and enhanced sensitivity to chemotherapeutic agents to decrease cancer cell viability.

SCF-Skp2 coordinates with Cks1 to promote the proliferation of cancer cells, via promoting the breakdown of p27, a CDK inhibitor (Tian et al., 2013). Wu et al. (2012) targeted the p27-binding interface within the Skp2-Cks1 complex using *in silico* screening to discover selective Skp2 inhibitors. ICM-PocketFinder was used to find a site formed by both Skp2 and Cks1 within the Skp2-Cks1-p27 crystal structure. The pocket’s area and volume was estimated to be suitable for small molecule binding. A virtual ligand screening of 315,000 compounds using ICM-VLS generated 202 screening hits with binding energy less than –30 U, and 96 of those ligands were chosen based on Lipinski properties to eliminate non-druglike molecules. These hits were experimentally screened using and four active compounds (**7–10**) were obtained. These hits selectively reduced p27 degradation regulated by Skp2 through inhibiting p27 binding. In tumor cells, these molecules increased p27 accumulation in a Skp2-dependent manner and promoted cell-type-specific blockages at the G1 or G2/M stages. The compound also raised both p27 protein level and longevity in metastatic melanoma cells, in a manner dependent on Skp2.

### Identification of Proteasome Regulators by Virtual Screening

Substrates modified by a polyubiquitination chain will be degraded by the 26S proteasome (Marshall and Vierstra, 2019). The 26S proteasome is a multi-subunit protease comprised of the catalytic core 20S proteasome and 19S regulatory subunits. The 20S catalytic core has a barrel structure and consists of four heptameric rings. On the outside are two identical non-catalytic α rings, and in the middle are two identical catalytic β rings. The 19S regulatory subunit has a structure comprised of a lid and a base. The 20S catalytic core of 26S proteasome is an important research target for proteasome inhibitors (Manasanch and Orlowski, 2017).

Di Giovanni et al. (2016) used a hierarchical screening approach for the 65,000 NCI lead-like library, followed by similarity searching over the entire NCI database using the most potent hit, in order to identify the β5/β6-specific tripeptide derivative **11** as a selective inhibitor of chymotrypsin-like proteasome activity. Specifically, three *in silico* screening modules (FRED, GLIDE, and GOLD) were used. Flexible docking was conducted using default parameters with GLIDE Standard Precision and Extra Precision, generating a shortlist of 500 top-ranked compounds. These molecules were re-docked using GOLD, then scored using the ChemPLP scoring function. For hit selection, two distinct hit lists were produced from the GLIDE and GOLD-ranked molecules. Manual inspection of binding followed by pharmacokinetics and PAINS filtering by FAF-Drugs2 resulted in a top list of 33 molecules, which were tested in an *in vitro* assay against the 20S proteasome. Finally, scaffold searching using the most active hit identified the most promising compound **11**, which showed an antiproliferative IC_50_ value of 16.2 ± 1.8 μM against multiple myeloma MM.1R cells.

Miller et al. (2015) conducted large-scale SBVS of small molecules at the active sites of the proteasome. The proteasome conformation was docking was prepared by molecular dynamics simulation of homology models with highly potent peptide ligands. By considering binding energy and interactions of known ligands at the binding site, the proteasome conformation at 341 ps was chosen for docking. After docking over 345,000 molecules from the University of Cincinnati database, 288 ligands were shortlisted by consensus scoring using various force field-based energy scoring functions (MM-PBSA and MM-GBSA), as well as visual inspection of binding poses. Of the tested molecules, 19 compounds were active at 5 μM in a CT-L activity assay. Subsequent optimization led to the identification of a non-peptide, reversible proteasome inhibitor compound **12** (G4-1) which showed an IC_50_ value of 10.5 μM against human pancreatic cancer BxPC-3 cells. The compound also showed excellent metabolic stability and effectively suppressed prostate cancer growth in a mice xenograft model without apparent systemic toxicity.

### Identification of Deubiquitinating Enzyme Regulators by Virtual Screening

DUBs can reverse the ubiquitination process through opposing the activity of E3 ligases, then saving the substrate from proteasomal degradation. Hence, DUB activity is critical for regulating physiological events such as cell growth and differentiation, transcription regulation, but its dysfunction can also lead to oncogenesis.

Ubiquitin-specific protease 7 (USP7) is one of the most widely studied and characteristic DUBs, and is a potential cancer treatment target. Through structure-based screening, molecular dynamics simulation and experimental valuation evaluation, Liu S. et al. (2020) discovered compound **13** as a new scaffold structure as an inhibitor of USP7. The Specs database of 250,000 compounds was screened using high-throughput virtual screening, standard-precision, and extra-precision modules in sequence. The top 10% of poses at each stage were shortlisted using Glide Gscore. The root-mean-square deviation and fluctuation metrics, which are related to complex stability, were calculated using the cpptraj module in AmberTools 15. Hydrogen bond occupancy, close contacts, equilibrated trajectories, and binding free energy (ΔGbind) were calculated using MM/PBSA. Finally, the highest-ranking 13 molecules were purchased, from which compound **13** was identified as the most potent hit after biological evaluation. The binding affinity between the USP7 catalytic domain and compound **13** was determined to be *K*_*d*_ = 4.46 ± 0.86 μM. This compound also showed an IC_50_ value of 15.43 ± 3.49 μM against LNCaP prostate cancer cells.

UCHL-3 (ubiquitin-C-terminal hydrol3) is a DUB involved in the homologous recombination repair mechanism of DNA double-strand breaks (DSBs). A number of studies have shown that UCHL-3 inhibitors can be used in combination therapy for treating cancer. Alakhdar et al. (2020) utilized a combination of virtual screening methods and 3D structures of more than 1.8 million compounds from the ChemBl database for virtual screening. Their strategy involved the combination of Lipinski’s Rule of Five, hierarchical molecular docking, pharmacophore modeling, toxicity and PAINS filter, Veber’s rule, and single-point molecular mechanics Poisson/Boltzmann surface area docking pose rescoring (Ma et al., 2011). This multiple filtering approach resulted in the shortlisting of 21 ligands, which were then analyzed using molecular dynamics simulations to estimate complex stability. Then, MM/PBSA calculations were performed on MD trajectories to calculate the energy per residue contribute to the binding energy. A 3D pharmacophore model was generated for identifying the significant features of reported UCHL3 inhibitors. Finally, three new potential UCHL-3 inhibitors (compounds **14–16**) were identified.

## Conclusion

Ubiquitination is a key regulatory process in cells, and is also a target for cancer treatment. Meanwhile, virtual screening has emerged as an indispensable part of drug discovery efforts and has become widely employed in pharmaceutical research. In this review, we have highlighted the use of virtual screening strategies to identify ubiquitination regulators, including E2, E3, proteasome and DUB regulators. Most of these studies have used SBVS, while LBVS has not been employed as much for the screening of ubiquitination regulators by comparison.

The main challenge facing virtual screening is still limited accuracy regardless using of the SBVS or LBVS methods. While calculations can be performed quickly relative to biological experiments, the false positive rate is still relatively high. Therefore, it is necessary to continuously improve the screening and scoring methods, or utilize novel techniques, such as predictive algorithms derived from machine and/pr deep learning, in order to improve the hit rate from virtual screening. Toward the future, one aspect that has significant room for enhancement in virtual screening is the prediction of potency. A key advancement would be if more consistent approaches could be developed for identifying more effective and druglike ligands *in silico*, thus directly benefiting subsequent drug discovery steps.

## Author Contributions

Y-QS drafted the manuscript of this review article. D-LM and C-HL conceived and supervised the manuscript and approved the final version to be published. CW, K-JW, Q-BH, and X-MM contributed to the conceptualization and provided critical responses to reviewers’ comments. CW and K-JW also collected and organized the information and prepared the table and figures for the revised manuscript. All authors contributed to the article and approved the submitted version.

## Conflict of Interest

The authors declare that the research was conducted in the absence of any commercial or financial relationships that could be construed as a potential conflict of interest.
